# Supplementary material for: Mechanism‐Driven Screening of Membrane‐Targeting and Pore‐Forming Antimicrobial Peptides
Source: Adv Sci (Weinh). 2025 Dec 14;13(9):e16470. doi: 10.1002/advs.202516470 (PMC12904036; doi:10.1002/advs.202516470)
Supplement: Supplementary file 1 — Supporting Information [file ADVS-13-e16470-s001.pdf]

Supplementary Information for:

Mechanism-driven screening of membrane-targeting and pore-forming antimicrobial peptides

Jiaxuan Li[1,2,†], Chenguang Yang[3,4,†], Ruihan Dong[1], Juan Francisco Bada Juarez[5], Lei Wang[1], Maximilian Emanuel Wettstein[5], Dali Wang[1], Chan Cao[6], Ying Lu[3,4,7,*], and Chen Song[1,*]

[1]Center for Quantitative Biology, Peking-Tsinghua Center for Life Sciences, Academy for Advanced Interdisciplinary Studies, Peking University, Beijing 100871, China

[2]College of Life Science, Yantai University, Yantai 264005, China

[3]Beijing National Laboratory for Condensed Matter Physics, Institute of Physics, Chinese Academy of Sciences, Beijing 100190, China

[4]University of Chinese Academy of Sciences, Beijing 100049, China

[5]Institute of Bioengineering, School of Life Sciences, École Polytechnique Fédérale de Lausanne (EPFL), 1015 Lausanne, Switzerland

[6]Department of Inorganic and Analytical Chemistry, University of Geneva, 1211 Geneva, Switzerland

[7]Songshan Lake Materials Laboratory, Dongguan, Guangdong 523808, China

[†]These authors contributed equally.

[*]E-mail: yinglu@iphy.ac.cn (YL), c.song@pku.edu.cn (CS)

1 Supplementary Tables

Table S1: 10-fold cross-validations of our SVM predictor.

Fold	Sensitivity	Specificity	Accuracy	Precision	MCC	F1	AUROC
1	0.9820	0.9918	0.9910	0.9213	0.9463	0.9507	0.9869
2	0.9671	0.9913	0.9894	0.9074	0.9311	0.9363	0.9792
3	0.9800	0.9925	0.9915	0.9188	0.9443	0.9484	0.9862
4	0.9607	0.9894	0.9867	0.9048	0.9250	0.9319	0.9751
5	0.9706	0.9907	0.9888	0.9116	0.9346	0.9402	0.9806
6	0.9627	0.9925	0.9899	0.9226	0.9370	0.9422	0.9776
7	0.9477	0.9936	0.9894	0.9368	0.9364	0.9422	0.9706
8	0.9481	0.9936	0.9899	0.9299	0.9335	0.9389	0.9708
9	0.9467	0.9907	0.9867	0.9091	0.9205	0.9275	0.9687
10	0.9551	0.9896	0.9867	0.8922	0.9160	0.9226	0.9724
**average**	0.9621	0.9916	0.9890	0.9155	0.9325	0.9381	0.9768
**std**	0.0123	0.0014	0.0017	0.0124	0.0092	0.0084	0.0061

Table S2: Benchmarking performances of various models using validation dataset. Best performances are shown in bold.

Year	Method	Model	Sensitivity	Specificity	Accuracy	Precision	MCC	F1	AUROC
2018	AMPScannerV2[1]	CNN+LSTM	0.7860	0.8706	0.8283	0.8587	0.6590	0.8212	0.9010
2020	MACREL[2]	RF	0.7799	0.8670	0.8235	0.8543	0.6494	0.8160	0.8932
2021	AmPEPpy[3]	RF	0.7050	0.8948	0.7999	0.8701	0.6109	0.7738	0.8741
2022	AMPlify[4]	LSTM+CNN	0.7789	**0.9033**	0.8416	**0.8897**	0.6884	0.8307	0.9069
2022	AMP-BERT[5]	BERT+MLP	0.7993	0.8756	0.8374	0.8652	0.6767	0.8309	0.8374
2023	Cao et al.[6]	BERT+MLP	0.8186	0.8875	0.8531	0.8792	0.7079	0.8471	**0.9141**
2023	AMPpred-MFA[7]	LSTM+CNN+Attention	0.7545	0.8887	0.8216	0.8715	0.6492	0.8088	0.8942
	Ours	BERT+SVM	**0.8247**	0.8948	**0.8597**	0.8869	**0.7212**	**0.8546**	0.8597

__: second-best performances

Table S3: Benchmarking MCP and secondary structure (SS) prediction criteria with labeled sequences in DBAASP. H: α-helix, E: β-sheet, C: coil.

Sequence	Length	MCP	SS
RVKRVWPLVIRTVIAGYNLYRAIKKK	26	>0.8	H
VRRFPWWWPFLRR	13	>0.7	C
SMWSGMWRRKLKKLRNALKKKLKGE	25	>0.6	H
GLFGKLIKKFGRKAISYAVKKARGKH	26	>0.5	H
RSVCRQIKICRRRGGCYYKCTNRPY	25	0.01	HCE
GIGGKILSGLKTALKGAAKELASTYLH	27	>0.4	H
GSKKPVPIIYCNRRTGKCQRM	21	0.09	CE
HVDKKVADKVLLLKQLRIMRLLTRL	25	>0.6	H
DTHFPICIFCCGCCHRSKCGMCCKT	25	>0.6	CE
KWCFRVCYRGICYRRCR	17	0.01	CE
GAWKNFWSSLRKGFYDGEAGRAIRR	25	>0.8	HC
GLNALKKVFQGIHEAIKLINNHVQ	24	>0.4	H
KSSAYSLQMGATAIKQVKKLFKKWGW	26	>0.3	H
FLPILASLAAKFGPKLFCLVTKKC	24	>0.9	H
FLPKLFAKITKKNMAHIR	18	>0.8	H
GRGREFMSNLKEKLSGVKEKMKNS	24	0.03	H
VGECVRGRCPSGMCCSQFGYCGKGPKYCG	29	0.08	C
KRFWPLVPVAINTVAAGINLYKAIRRK	27	>0.8	H
RKCNFLCKLKEKLRTVITSHIDKVLRPQG	29	>0.2	H
RGGGLCYCRRRFCVCVGR	18	0.04	CE
RGGRLCYCRGWICFCVGR	18	>0.2	CE
RGGRLCYCRPRFCVCVGR	18	0.07	CE
FFHHIFRGIVHVGKTIHRLVTG	22	>0.7	H
GIGAVLKVLTTGLPALISWIKRKRQQ	26	>0.8	H
FIHHIIGGLFSAGKAIHRLIRRRRR	25	>0.7	H
LLPIVGNLLKSLL	13	>0.8	H
RVKRFWPLVPVAINTVAAGINLYKAIRRK	29	>0.8	H
FLPLIGRVLSGIL	13	>0.9	H
NLCASLRARHTIPQCRKFGRR	21	>0.5	H
GLKEIFKAGLGSLVKGIAAHVAS	23	>0.8	H

Table S4: Statistics of the sequence number in the screening process. SVM predictor and anti-parallel dimer CM prediction are rate-determining steps, where very few sequences could meet the expectations of mechanisms.

Metaproteome source	Initial sequences	AMP classification			Post-classification		
		Pass MCP	Pass SS	Pass SVM	Positive charge	BLAST-p	Anti-parallel dimer (CM)
**Poison frog**	331689	119013	51573	**268 (0.5%)**	227	226	**11 (4.9%)**
**African clawed frog**	999997	399781	189424	**3250 (1.7%)**	2986	2956	**67 (2.3%)**
**Human skin**	101386	36475	15968	**75 (0.5%)**	56	56	**2 (3.6%)**

Table S5: Sequences, physicochemical properties, and the predicted perforated ability (F_{score}) of 20 selected antimicrobial peptides. Two sequences with underline are too hydrophobic for chemical synthesis.

Peptide name	Sequence	Charge	Length	Hydrophilicity	F_{score}	E-value
1422	SKSVALGILSALGAVGLAVGGVIAMGVLGDK	+1	31	0.10	0.667	2.9
661	TVLASIVGLVGLLVTIVGAKCTNCLQGS	+1	28	0.10	0.635	3.7
163	FRLALLGMHLICLALGVLR	+3	19	0.16	0.592	–
4	FAAAAIGSHALTFLKRAR	+4	18	0.22	0.501	–
98	GPIHRLINPFFNLLQGIFTN	+2	20	0.30	0.396	5.5
30	LNTFSPLAAALHIIEK	+1	16	0.25	0.341	–
136	AVGAAAVGAVSSFIAYQKK	+2	19	0.16	0.333	–
68	GFKSKLEQISGICRPLFAAMWK	+3	22	0.27	0.332	8.7
1304	IYKILQLFHKRFKKGFFG	+6	18	0.39	0.328	–
171	ALTALINGGLSHLNTAVK	+2	18	0.22	0.328	9.2
239	QAKGFGSYLLNFASVATKKLS	+3	21	0.24	0.324	9.9
58	KVKVLIIRKLTRHIAKLKSKK	+10	21	0.48	0.319	9.3
623	KLAKLLKLLLWAQNELDQKK	+3	20	0.50	0.312	–
615	KKIASIIYAHVKALRARKILDNLKRLAANRP	+9	31	0.42	0.292	7.1
162	GINTACRGIAQISAAVFDK	+1	19	0.26	0.282	–
986	FVFLIMALFEAIVARWFAKKKLES	+2	24	0.25	0.273	–
201	AKLVALVNKFIGYLKQNTYC	+3	20	0.30	0.264	7.8
1248	DPFQALNRVTLPCKKYFVRVSVLAICAGV	+3	29	0.24	0.262	–
173	NNWGIGLGVLVREYGK	+1	16	0.31	0.253	–
737	NLLRKRRSGLLPLLIRS	+5	17	0.35	0.252	–

Table S6: Hemolysis tests on rat blood cell (RBC). Both EC_{50} and MHC are larger than 128 μg/mL for all seven peptides. MHC: the lowest concentration of peptide that causes 10% hemolysis. Positive control : 10 mg/mL Triton X-100 + RBC with 100% hemolysis.

**Compounds**	**EC_{50} (μg/mL)**	**MHC (μg/mL)**
58	>128	>128
68	>128	>128
98	>128	>128
201	>128	>128
615	>128	>128
623	>128	>128
1304	>128	>128

Table S7: Cytotoxicity tests of seven peptides on HEK293T cells. CC_{50} is larger than 128 µg/mL for all screened peptides. Controls: Amphotericin B and Staurosporine.

**Compounds**	**HEK293T** CC_{50} (µg/mL)
58	>128
68	>128
98	>128
201	>128
615	>128
623	>128
1304	>128
Amphotericin B	67.06
Staurospotine	0.26

2 Supplementary Figures

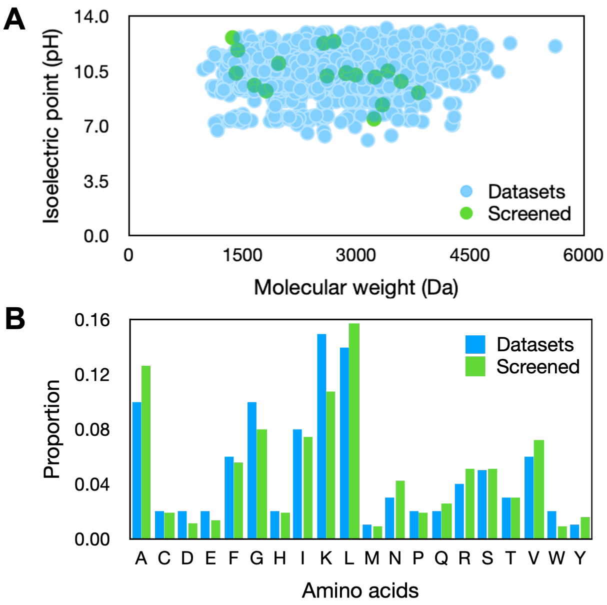

Figure S1: Charecteristic comparison between positive dataset and 20 screened peptides. (A) Distribution of isoelectric point and molecular weight. (B) Distribution of amino acids.

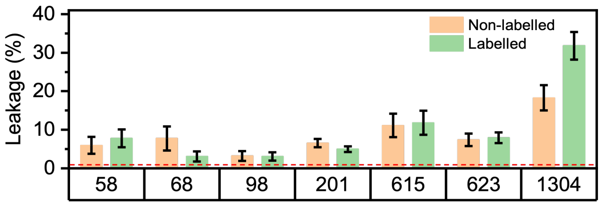

Figure S2: Leakage of carboxyfluorescein (CF) from liposomes induced by peptides and fluorophores-labeled peptides. Histogram represents the leakage of CF from liposomes after the addition of peptides, and is shown as a percentage of that induced by the addition of detergent 0.2% Triton X-100. Red dashed lines indicate the background leakage from the liposomes incubated with an equivalent concentration of buffer alone, to control for the effect of buffer. Data are presented as mean ± SEM (n = 3 independent measurements).

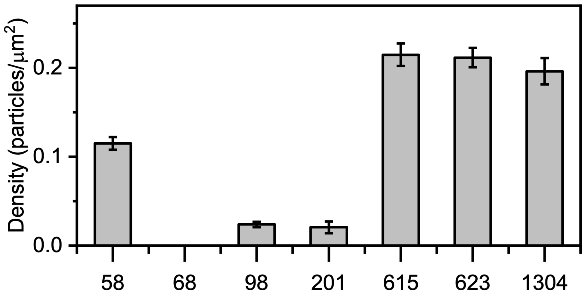

Figure S3: Surface density of peptides absorbing on lipid bilayer. Data are presented as mean ± SEM (n = 3 independent measurements).

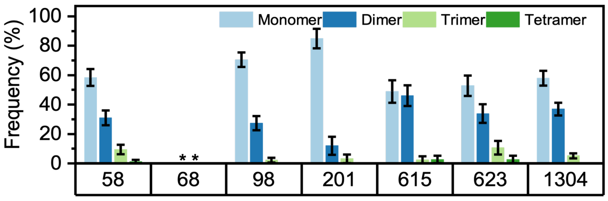

Figure S4: Oligomers distribution of peptides on supported lipid bilayers with washing free peptides in solution. Histogram of photobleaching step numbers for fluorescence labelled peptides analyzed. Asterisks represent the data of peptide 68 was not further analyzed. Data are presented as mean ± SD. Error bars correspond to the bootstrapped standard deviation.

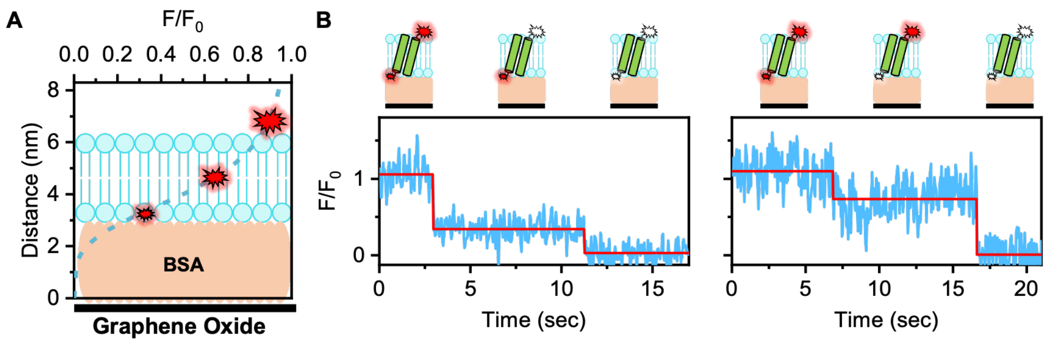

Figure S5: Detecting membrane topology of dimeric peptide 201 by smSIFA (single molecule surface-induced fluorescence attenuation). (A) The dependence of the intensity ratio on the distance to the surface of the single layer graphene oxide in smSIFA. (B) Two representative intensity ratio traces (intensity trace, blue; idealized fitting curves, red) of antiparallel dimeric peptide 201.

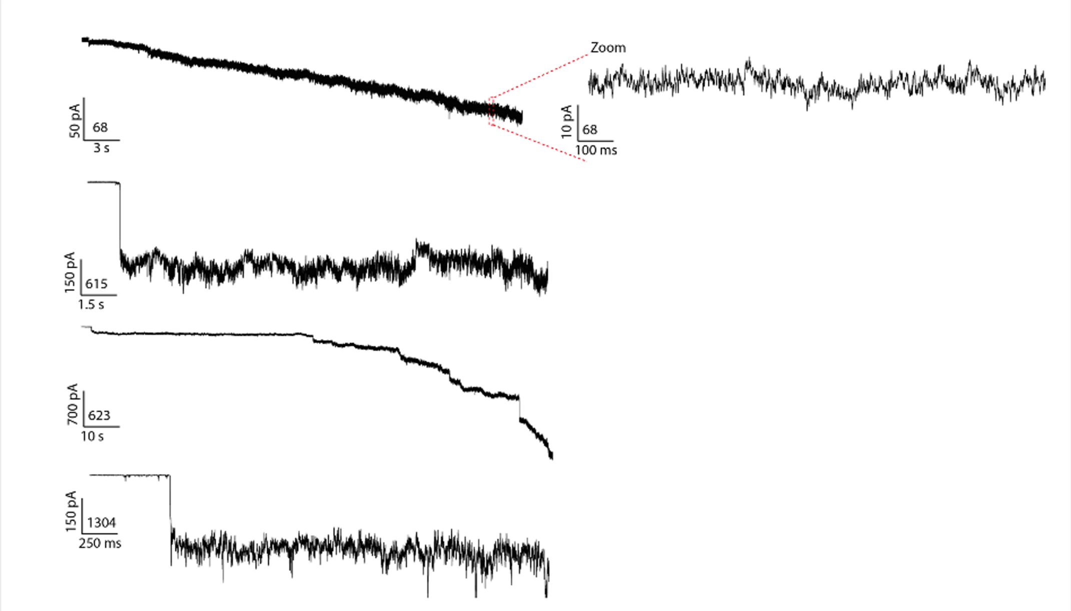

Figure S6: Single-channel bilayer experiments results for peptide 68, 615, 623, and 1304. These traces show disturbance of the peptide while in contact with the membrane. Peptides 615, 623 and 1304 show clear step indicative for pore insertion. For peptide 68, we do observe disturbance of the membrane with no clear steps like in the other peptides.

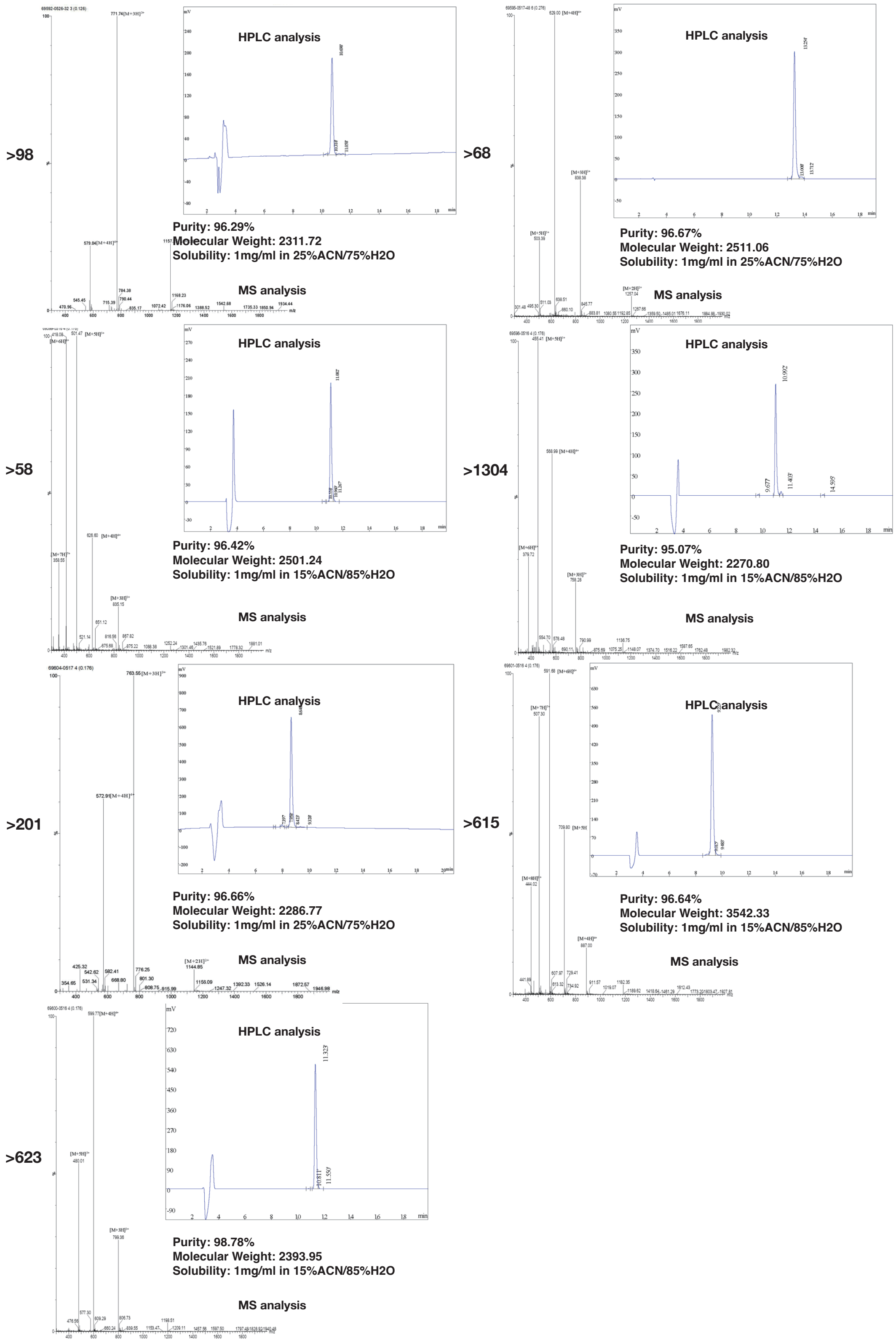

Figure S7: Synthesis reports of 7 effective peptides.

References

[1] Veltri, D.; Kamath, U.; Shehu, A. Deep learning improves antimicrobial peptide recognition. *Bioinformatics* **2018**, *34*, 2740–2747.

[2] Santos-Júnior, C. D.; Pan, S.; Zhao, X. M.; Coelho, L. P. Macrel: Antimicrobial peptide screening in genomes and metagenomes. *PeerJ* **2020**, *8*.

[3] Lawrence, T. J.; Carper, D. L.; Spangler, M. K.; Carrell, A. A.; Rush, T. A.; Minter, S. J.; Weston, D. J.; Labbé, J. L. amPEPpy 1.0: a portable and accurate antimicrobial peptide prediction tool. *Bioinformatics* **2020**, *37*, 2058–2060.

[4] Li, C.; Sutherland, D.; Hammond, S. A.; Yang, C.; Taho, F.; Bergman, L.; Houston, S.; Warren, R. L.; Wong, T.; Hoang, L. M.; Cameron, C. E.; Helbing, C. C.; Birol, I. AMPlify: attentive deep learning model for discovery of novel antimicrobial peptides effective against WHO priority pathogens. *BMC Genomics* **2022**, *23*.

[5] Lee, H.; Lee, S.; Lee, I.; Nam, H. AMP-BERT: Prediction of antimicrobial peptide function based on a BERT model. *Protein Science* **2023**, *32*.

[6] Cao, Q. et al. Designing antimicrobial peptides using deep learning and molecular dynamic simulations. *Briefings in Bioinformatics* **2023**,

[7] Li, C.; Zou, Q.; Jia, C.; Zheng, J. AMPpred-MFA: An Interpretable Antimicrobial Peptide Predictor with a Stacking Architecture, Multiple Features, and Multihead Attention. *Journal of Chemical Information and Modeling* **2024**, *64*, 2393–2404.
